# Understanding Chronic Hip Pain Through the ICF Framework: Implications for Assessment, Diagnosis, and Management

**DOI:** 10.1111/os.70416

**Published:** 2026-10-08

**Authors:** Mingke You, Kai Zhou, Xi Chen, Xue Zhang, Jian Li, Gang Chen

**Affiliations:** ^1^ Sports Medicine Center, Department of Orthopedics and Orthopedic Research Institute West China Hospital, West China School of Medicine, Sichuan University Chengdu City Sichuan Province China

**Keywords:** activity and participation, chronic hip pain, chronic musculoskeletal pain, ICF, rehabilitation

## Abstract

Chronic hip pain is a prevalent musculoskeletal condition that can impair mobility, physical function, participation, and quality of life. Traditional assessment and management approaches often emphasize structural abnormalities and symptom control, but these approaches may not fully capture the broader functional, psychosocial, and contextual impact of chronic hip pain. The International Classification of Functioning, Disability and Health (ICF) provides a multidimensional framework for understanding health conditions through body structures and functions, activity and participation, and contextual factors. This scoping review aimed to synthesize recent evidence on chronic hip pain using the ICF framework and to clarify how this framework may inform assessment, diagnostic reasoning, and treatment planning. A literature search was conducted in PubMed, Embase, and Ovid for studies published between January 1, 2020, and December 31, 2025. Original studies related to chronic hip pain or hip‐related chronic pain were included and classified according to ICF‐related domains, including structures and functions, activity and participation, personal factors, and environmental factors. A total of 39 studies were included. A total of 19 studies, accounting for 48.7% of the included literature, were classified under structures and functions. These studies mainly addressed structural abnormalities, pain mechanisms, clinical assessment, pharmacological treatment, interventional procedures, and other symptom‐oriented interventions. A total of 11 studies were classified under activity and participation, focusing on movement performance, functional activities, physical activity, quality of life, and community‐based outcomes. Seven studies examined personal factors, including psychological status, sleep quality, opioid use, race, and pain management strategies. Only two studies addressed environmental factors, mainly involving community‐based care and online education. Overall, current research on chronic hip pain remains predominantly focused on body‐level impairments and symptom management, whereas participation‐level outcomes and environmental factors are comparatively underrepresented. The ICF framework offers a useful structure for integrating structural findings with functional limitations, psychosocial factors, and contextual influences. Applying this framework may support more comprehensive assessment, clinically meaningful diagnostic reasoning, and individualized treatment planning for people with chronic hip pain. Future studies should place greater emphasis on participation and environmental factors to better inform patient‐centered rehabilitation and long‐term management.

## Graphical abstract

Chronic hip pain remains predominantly studied from the perspective of body‐level impairments and symptom management. Using the International Classification of Functioning, Disability and Health (ICF) framework, this review synthesized evidence published between 2020 and 2025 and found that most studies focused on structures and functions, while activity and participation, personal factors, and environmental factors were less frequently addressed. The ICF framework helps integrate structural findings with functional limitations, psychosocial influences, and contextual factors, supporting more comprehensive assessment, clinically meaningful diagnostic reasoning, and individualized treatment planning. Greater attention to participation and environmental factors may improve patient‐centered rehabilitation and long‐term management.

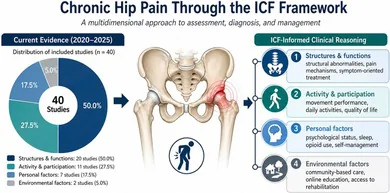

## 1 Introduction

Chronic hip pain is commonly defined as pain persisting for more than three months and may arise from a wide range of conditions, including intra‐articular hip pathology, periarticular soft‐tissue disorders, referred pain from adjacent regions, trauma, or systemic disease [1, 2]. It is frequently associated with restricted hip range of motion, reduced muscle performance, altered movement patterns, impaired mobility, and decreased quality of life. Epidemiological studies suggest that hip pain affects approximately 10% of the general population, and up to one‐third of adults over 50 years report hip discomfort [3, 4]. Therefore, chronic hip pain represents not only a local musculoskeletal symptom but also a condition with substantial functional and social consequences.

Traditionally, the assessment and diagnosis of chronic hip pain have focused primarily on anatomical pathology and structural abnormalities identified through imaging, clinical examination, and disease‐specific classification [5]. Although this approach is essential for identifying potential sources of pain, it may not fully explain the lived experience of patients with chronic hip pain. In clinical practice, structural findings may not always correspond to pain severity, functional limitation, or treatment response. This discordance has been demonstrated in hip pain populations; for example, a large BMJ study reported that many individuals with radiographic hip osteoarthritis did not report hip pain, while many patients with hip pain showed no radiographic evidence of hip osteoarthritis. Therefore, some patients may present with significant pain and disability despite limited radiological abnormalities, whereas others may show marked structural changes with relatively mild symptoms [6]. This mismatch highlights the need for a broader framework that integrates structural pathology with functional performance, activity limitation, psychological status, and contextual influences.

The International Classification of Functioning, Disability and Health framework, developed by the World Health Organization, provides a biopsychosocial model for describing health and disability [7]. Unlike disease‐based classification systems, the ICF framework considers health conditions through multiple interacting domains, including body functions and structures, activity and participation, personal factors, and environmental factors [8, 9]. This multidimensional perspective is particularly relevant to chronic hip pain because patients often experience pain and physical impairment, as well as limitations in walking, stair climbing, work participation, physical activity, self‐management, and psychosocial well‐being.

Previous studies have applied the ICF framework to several musculoskeletal conditions, including knee osteoarthritis, shoulder pain, and broader musculoskeletal disorders, demonstrating its usefulness in describing impairments, activity limitations, and participation restrictions [9, 10, 11]. However, evidence specifically examining chronic hip pain through the ICF framework remains limited and fragmented. Existing studies often focus on isolated aspects such as imaging findings, surgical outcomes, gait characteristics, pain severity, or psychological factors, without integrating these findings into a comprehensive functional model.

Despite these applications, few studies have specifically focused on symptoms like chronic hip pain within the ICF framework. Amy et al. compared the Harris Hip Score with the ICF model, revealing significant overlap in activity and participation assessment (71% in the Harris Hip Score versus 82% in the ICF model) [12]. Two additional studies evaluated hip osteoarthritis with joint replacement and gait mechanisms using the ICF model, both confirming its effectiveness for assessment and treatment planning [13, 14].

Therefore, this scoping review aims to classify and synthesize existing literature on chronic hip pain according to the ICF framework. More importantly, it seeks to clarify how the ICF model may support multidimensional assessment, comprehensive diagnosis, and individualized treatment planning for people with chronic hip pain.

## 2 Methods

### 2.1 Search Strategy

A systematic literature search was conducted in PubMed, Embase, and Ovid databases to identify studies published between 1st of January 2020 and 31st of December 2025. The review was conducted and reported in accordance with the Preferred Reporting Items for Systematic Reviews and Meta‐Analyses Extension for Scoping Reviews (PRISMA‐ScR). The search strategy combined terms related to the hip joint and chronic pain. The following search terms were used: “((((((((hip joint) OR (Hip Joints)) OR (Joint, Hip)) OR (Joints, Hip)) OR (Acetabulofemoral Joint)) OR (Acetabulofemoral Joints)) OR (Joint, Acetabulofemoral)) OR (Joints, Acetabulofemoral)) AND (((((((((((chronic pain) OR (Pain, Chronic)) OR (Widespread Chronic Pain)) OR (Chronic Pain, Widespread)) OR (Pain, Widespread Chronic)) OR (Chronic Primary Pain)) OR (Pain, Chronic Primary)) OR (Primary Pain, Chronic)) OR (Chronic Secondary Pain)) OR (Pain, Chronic Secondary)) OR (Secondary Pain, Chronic))”.

### 2.2 Eligibility Criteria

Studies were eligible if they met the following criteria: 1. original research articles; 2. studies involving adults or mixed populations with chronic hip pain or hip‐related chronic pain; 3. studies reporting assessment, diagnosis, treatment, prognosis, functional outcomes, activity limitations, participation restrictions, or contextual factors related to chronic hip pain; 4. studies published in English.

Reviews, case reports, book chapters, conference abstracts, reference papers, product descriptions, non‐English articles, and studies not directly related to chronic hip pain were excluded.

### 2.3 Study Selection

All retrieved records were screened according to title and abstract. Articles that appeared relevant were then assessed in full text. Studies that met the inclusion criteria were included in the final analysis. The database search identified 1624 records. After removing 508 duplicate records, 1116 records underwent title screening, and then 886 records underwent abstract screening. Subsequently, 60 full‐text articles were assessed for eligibility, of which 21 articles were excluded. Finally, 39 studies were included in this scoping review. The screening and inclusion process is presented in Figure 1.

**FIGURE 1:**
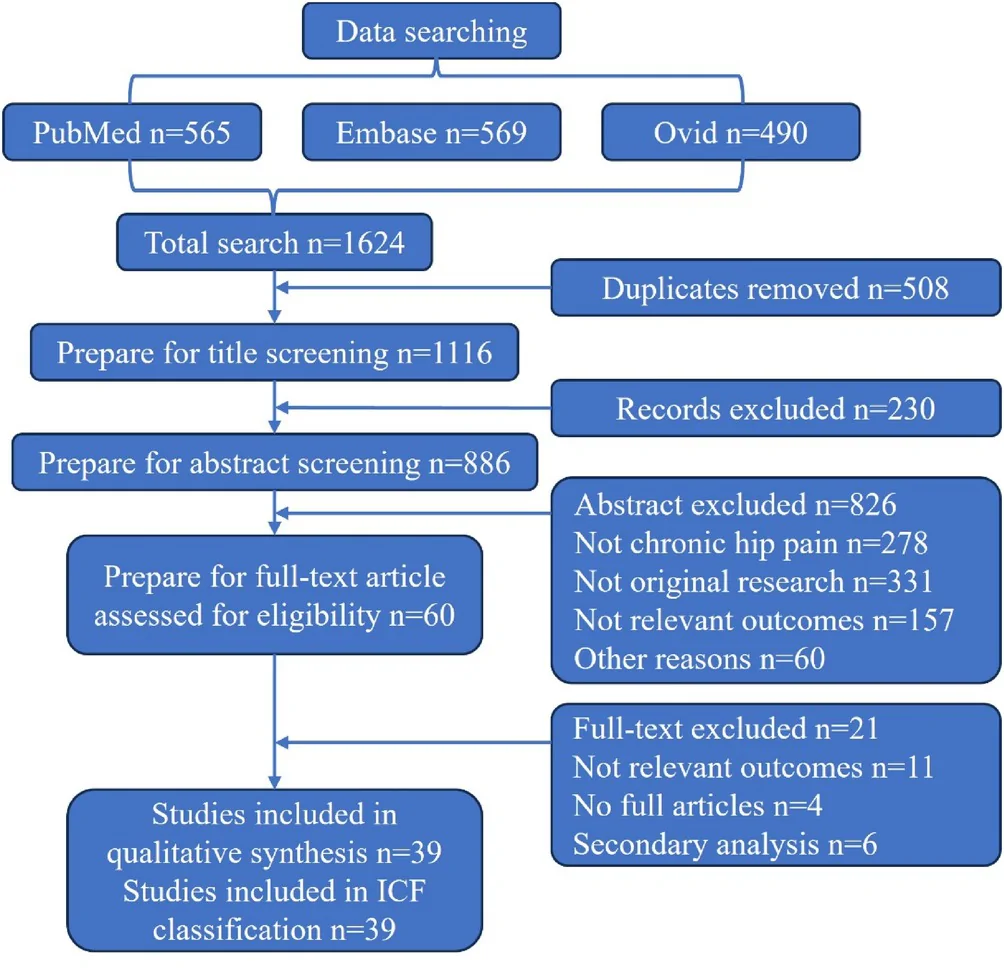
A flow chart of the screening and inclusion process.

### 2.4 ICF‐Based Classification

Included studies were classified according to the core components of the International Classification of Functioning, Disability and Health framework. Two reviewers (YMK and ZK) independently classified each study according to the ICF framework; detailed classifications were listed below. The classification included the following domains:

1. Body structures and body functions: studies focusing on anatomical abnormalities, imaging findings, pain mechanisms, muscle strength, range of motion, physical impairments, clinical diagnosis, or interventions targeting pain and physical dysfunction.
2. Activity: studies examining movement performance, gait, walking ability, stair climbing, squatting, functional mobility, exercise performance, or self‐management activities.
3. Participation: studies reporting work status, social participation, community activity, quality of life, or restrictions in social roles related to chronic hip pain.
4. Personal factors: studies assessing individual characteristics such as age, sex, body mass index, psychological status, sleep quality, pain catastrophizing, joint confidence, education, lifestyle, or self‐efficacy.
5. Environmental factors: studies examining contextual influences such as healthcare access, family or social support, workplace environment, community resources, assistive devices, or environmental modification.

When a study involved more than one ICF component, it was classified according to its primary research focus. If the study addressed multiple domains with similar emphasis, all relevant ICF components were recorded.

Studies involving mixed hip and knee populations were eligible when participants with chronic hip pain, hip osteoarthritis, or another hip‐related chronic pain condition were explicitly included. Hip‐specific findings were extracted when available. When hip and knee results were reported only in aggregate, the study was retained for descriptive ICF‐based evidence mapping, but its findings were not interpreted as hip‐specific effects.

### 2.5 Methodological Quality and Risk‐Of‐Bias Assessment

The methodological quality and risk of bias of the included studies were assessed according to study design. Randomized controlled trials were evaluated using the revised Cochrane Risk of Bias tool for randomized trials (RoB 2). Cluster‐randomized controlled trials were assessed separately using the RoB 2 tool for cluster‐randomized trials. Cohort studies were assessed using the Joanna Briggs Institute (JBI) Critical Appraisal Checklist for Cohort Studies. Analytical cross‐sectional studies were assessed using the JBI Critical Appraisal Checklist for Analytical Cross‐Sectional Studies.

Two reviewers (YMK and ZX) independently conducted the quality/risk‐of‐bias assessment for all included studies. For RoB 2, each randomized controlled trial was assessed across five domains: bias arising from the randomization process, bias due to deviations from intended interventions, bias due to missing outcome data, bias in measurement of the outcome, and bias in selection of the reported result. Each domain and the overall risk of bias were judged as “low risk,” “some concerns,” or “high risk.” For cluster‐randomized trials, additional considerations related to the timing of identification or recruitment of participants, baseline imbalances, loss of clusters or individuals, and comparability with individually randomized trials were also assessed.

For cohort studies, the JBI checklist assessed 11 items, including the comparability of groups, exposure measurement, identification and management of confounding factors, baseline outcome status, outcome measurement, adequacy and completeness of follow‐up, strategies for incomplete follow‐up, and appropriateness of statistical analysis. For analytical cross‐sectional studies, the JBI checklist assessed 8 items, including the clarity of inclusion criteria, description of participants and setting, validity and reliability of exposure and outcome measurements, use of objective criteria for condition measurement, identification and management of confounding factors, and appropriateness of statistical analysis. Each JBI item was rated as “yes,” “no,” “unclear,” or “not applicable,” followed by an overall appraisal.

Any disagreements between the two reviewers were resolved through discussion until consensus was reached. The results of the methodological quality and risk‐of‐bias assessments were summarized and incorporated into the interpretation of the reliability of the review findings.

### 2.6 Data Extraction and Synthesis

For each included study, the following information was extracted: author, year of publication, study design, population, hip pain condition, main assessment or intervention, outcome measures, and corresponding ICF domain. Studies were then grouped according to ICF components and further categorized by their clinical focus, including assessment, diagnosis, treatment, influencing factors, prognosis, activity limitation, participation restriction, and contextual factors.

A narrative synthesis was conducted to summarize how current evidence on chronic hip pain maps onto the ICF framework. Particular attention was paid to how each ICF component contributes to clinical assessment, diagnostic reasoning, treatment selection, and rehabilitation planning. Evidence gaps were identified by comparing the distribution of studies across ICF domains.

## 3 Results

### 3.1 General Characteristics

A total of 39 studies published between 2020 and 2025 were included in this review (Table 1). The included studies covered a range of hip‐related chronic pain conditions, including greater trochanteric pain syndrome, hip osteoarthritis, chronic pain after total hip arthroplasty, nonarthritic hip‐related pain, sickle‐cell‐disease‐related hip pain, and other hip‐related or adjacent‐region pain conditions. Study designs included randomized controlled trials, cohort studies, cross‐sectional studies, observational studies, comparative studies, and clinical trials. According to the ICF framework, 19 studies (19/39 = 48.7%) were classified under body structures and body functions, 11 under activity and participation, 7 under personal factors, and 2 under environmental factors (Figure 2).

**TABLE 1:** Information for included studies and their ICF domains.

Author	PMID	Year	Study design	Population
Canetti	31828381	2020	Case–control study	43
Ramon	32769596	2020	RCT	103
Hattori	33911897	2021	Cohort study	39
Blikman	35123461	2022	RCT	725
Driest	34989159	2022	Cluster‐randomized trial	132
Boljanovic‐Susic	37095584	2023	Cohort study	75
Crema	37720809	2023	RCT	44
Florencio	36657768	2023	Longitudinal study	250
Hah	37556071	2023	Cohort study	188
Correia	38465076	2024	Cohort study	26
Diwan	38558402	2024	Single‐arm clinical trial	31
Karaoğlan	38380579	2024	Retrospective study	112
Karaoğlu	38430008	2024	Cohort study	53
Petroni	38512631	2024	Observational study	44
Şirolu	39686471	2024	Comparative study	37
Akkemik	40523099	2025	Cohort study	58
Martin	40214220	2025	Cohort study	200
Laigaard	41072560	2025	Cross‐sectional study	2025
Pinto	40760428	2025	Cohort study	2025
Torrealba	40551997	2025	Observational study	30
Bilbao	32197734	2020	Observational study	313
Ferrer‐Peña	32839019	2020	Observational study	49
Vasiljevic	29963931	2020	Laboratory study	36
Skou	31492080	2020	Cross‐sectional study	3889
Smith	37180574	2023	Cohort study	1593
Lodin	34342204	2023	Comparative study	20
Lopes	36703210	2023	Cohort study	1086
Palaniappan	37939703	2023	RCT	30
Cheng	39953925	2025	Cohort study	88
Galassi	40426937	2025	Cohort study	Not mentioned for hip
Wainwright	40753994	2025	RCT	221
Wadström	36288602	2022	Cohort study	1292
George	34678465	2022	Observational study	1146
Hardy	35999466	2022	Observational study	105
Mezey	35268444	2022	Cross‐sectional study	189
Tang	37142067	2023	Observational study	80
Lopes	38060074	2024	Cross‐sectional study	24
Schwartz	41104353	2025	Cohort study	54
Hurley	28009618	2023	Cohort study	298
Nelligan	37189094	2023	RCT	120

Author	Condition	Main focus	Intervention/Outcome	ICF domains
Canetti	GTPS	Diagnosis	X‐ray finding	Structure & function
Ramon	GTPS	Treatment	F‐ESWT	Structure & function
Hattori	HOA	Diagnosis	NRS score	Structure & function
Blikman	HOA	Treatment	Duloxetine usage	Structure & function
Driest	HOA	Treatment	Duloxetine usage	Structure & function
Dragana	THA	Assessment	NP‐MPQ (SF‐2)	Structure & function
Crema	THA	Treatment	Lidocaine and phenol	Structure & function
Florencio	HOA	Assessment	Psychological&biomarker	Structure & function
Hah	THA	Treatment	opioid usage	Structure & function
Correia	HOA	Treatment	RFA	Structure & function
Diwan	THA	Treatment	PRF	Structure & function
Karaoğlan	HOA, GTPS, THA	Treatment	Ultrasound‐guided PENG block	Structure & function
Karaoğlu	HOA, THA, malignancy, avascular necrosis	Treatment	Radiofrequency thermocoagulation	Structure & function
Petroni	Hip pain	Treatment	Ultrasound guided ablation	Structure & function
Şirolu	GTPS	Diagnosis	SMI	Structure & function
Akkemik	Chronic sacroiliac joint pain	Treatment	Ultrasound‐guided pulsed radiofrequency	Structure & function
Martin	HOA	Treatment	Posterior quadratus lumborum block	Structure & function
Laigaard	THA	Assessment	WOMAC, DN4i, NRS	Structure & function
Pinto	Femur fracture	Assessment	Surgical access routes	Structure & function
Torrealba	THA	Treatment	PRF	Structure & function
Bilbao	HOA	Assessment	WOMAC and the EQ‐5D‐5L	Activity & participation
Ferrer‐Peña	GTPS	Activity limitation	SLST in abduction hip movements	Activity & participation
Vasiljevic	NA	Assessment	Pelvic kinematics and movement pattern	Activity & participation
Skou	HOA	Activity limitation	SLST in abduction hip movements	Activity & participation
Smith	HOA	Rehabilitation	12‐week program	Activity & participation
Lodin	SIJD	Activity limitation	Gait analysis	Activity & participation
Lopes	HOA	Participation restriction	Quality of life	Activity & participation
Palaniappan	NA	Rehabilitation	Movement pattern training	Activity & participation
Cheng	Nonarthritic hip‐related pain	Rehabilitation	Movement pattern modification	Activity & participation
Galassi	Hip pain	Rehabilitation	Vigorous physical activity	Activity & participation
Wainwright	HOA	Rehabilitation	Cycling and education	Activity & participation
Wadström	LCPD & SCFE	Self‐management	Depression	Personal
George	THA	Influencing factor	Race	Personal
Hardy	THA	Influencing factor	Pre‐operative psychological state	Personal
Mezey	HOA	Influencing factor	Pain management techniques	Personal
Tang	THA	Influencing factor	NRS & sleep disorders	Personal
Lopes	SCD	Assessment	Depression, anxiety	Personal
Schwartz	Hip pain	Influencing factor	Pain levels and patient‐reported outcomes	Personal
Hurley	Hip pain	Care pathway	Community‐based care	Environment
Nelligan	HOA	Care pathway	MOOC online course	Environment

Abbreviations: DN4i: Doleur Neuropatique 4 interview; EQ‐5D‐5L: EuroQol 5‐Dimension 5‐Level questionnaire; F‐ESWT: electromagnetic‐focused extracorporeal shockwave treatment; GTPS: Greater Trochanteric Pain Syndrome; HOA: hip osteoarthritis; LCPD: Legg‐Calvé‐Perthes disease; MOOC: Massive Open Online Course; NP‐MPQ: the Neuropathic Pain Subscale of McGill; NRS: Numerical Rating Scale; PENG: Pericapsular Nerve Group block; PRF: pulsed radiofrequency; RCT: randomized controlled trial; RFA: radiofrequency ablation; SCD: Sickle Cell Disease; SCFE: slipped capital femoral epiphysis; SIJD: Sacroiliac joint dysfunction; SLST: single‐leg‐squat test; SMI: Superb microvascular imaging; THA: total hip arthroplasty; WOMAC: Western Ontario and McMaster Universities Osteoarthritis Index.

**FIGURE 2:**
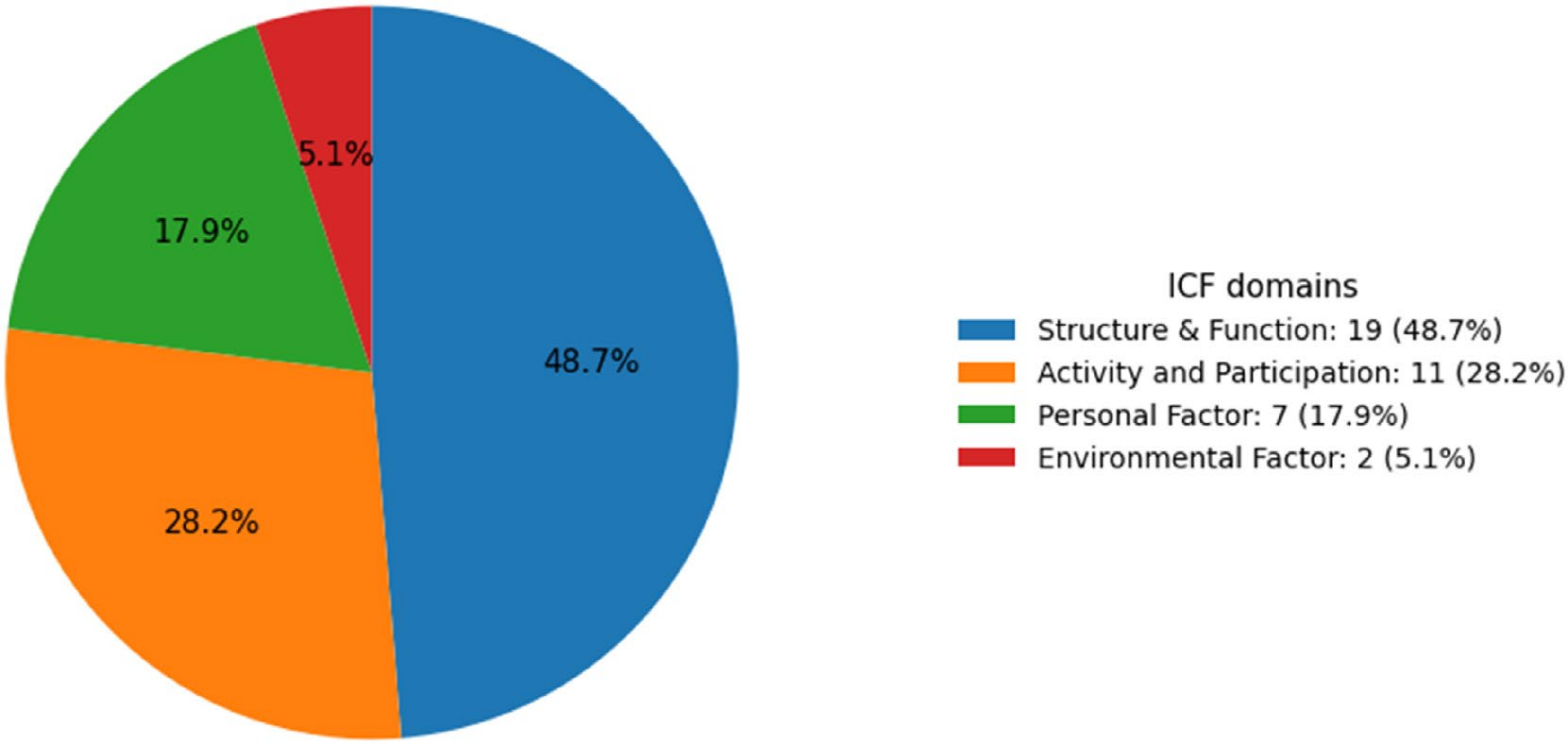
A pie chart of domains according to ICF framework.

### 3.2 Distribution of Studies in ICF Domains

The distribution of studies showed that current evidence on chronic hip pain remains concentrated in the domain of body structures and body functions, accounting for half of the included studies. Activity and participation represented the second largest category, whereas personal factors were less frequently investigated. Environmental factors were rarely addressed, with only two studies focusing on care pathways or digital/community‐based rehabilitation resources. This distribution suggests that although chronic hip pain has increasingly been examined beyond structural pathology, research remains uneven across ICF domains.

### 3.3 Structure and Functions

A total of 19 articles reported chronic hip pain from the perspective of structures and functions. These studies primarily focused on structural abnormalities, pain‐related dysfunction, and impairment‐oriented treatment in patients with chronic hip pain or hip‐related chronic pain. Imaging examinations [15, 16], clinical assessment scales [17, 18], pain questionnaires [19], medication usage [20, 21], and interventional techniques such as nerve blocks [22] were used to characterize body‐level impairments and guide symptom‐focused management.

Radiological assessment remained an important approach for evaluating chronic hip pain. Magnetic resonance imaging and ultrasound were mainly used to assess soft‐tissue or periarticular abnormalities, such as tendon pathology, muscle involvement, labral lesions, or chondral changes [23]. Computed tomography and X‐ray were more commonly used to evaluate skeletal morphology, osteoarthritis severity, spinopelvic parameters, or bony deformities [15, 19, 24]. In addition, these imaging‐based studies helped identify potential anatomical sources of pain and provided objective information for structural diagnosis.

In addition to structural imaging, several studies evaluated pain intensity, physical function, and pain mechanisms using clinical scales or psychophysical measures. Commonly reported outcomes included pain intensity, WOMAC, HOOS, NRS, DN4, NP‐MPQ, range of motion, muscle strength, pressure pain threshold, conditioned pain modulation, and central sensitization‐related measures [17, 18, 19, 25]. These assessments provided information on body functions that may not be fully captured by imaging findings alone.

Treatment‐related studies in this domain mainly focused on interventions targeting pain relief, physical impairment, or local pathological mechanisms. Interventional pain procedures included radiofrequency ablation [26], pulsed radiofrequency [27], ultrasound‐guided ablation [28], nerve blocks [22], and other injection‐based techniques [29]. These interventions primarily aimed to reduce pain intensity, decrease analgesic use, prolong pain relief, or improve hip‐related function by targeting articular branches, periarticular tissues, or pain‐generating structures. Pharmacological and physical treatment studies, such as duloxetine or extracorporeal shockwave therapy [20, 30, 31], also focused mainly on symptom modulation and improvement of body function.

In summary, the structures and functions domain represented the largest proportion of the included literature, accounting for 19 of 39 studies. This distribution indicates that chronic hip pain research remains largely centered on body‐level impairments, including structural pathology, pain intensity, pain mechanisms, and impairment‐oriented treatment. Nevertheless, 11 studies were mapped to activity and participation, showing that a smaller proportion of the literature has extended beyond body‐level impairment to examine movement performance, functional activities, and participation‐related outcomes. The evidence in this domain is presented in the next section.

### 3.4 Activity and Participation

A total of 11 articles were classified under the ICF domain of activity and participation, accounting for 28.2% of the included studies. Compared with studies focusing on structures and functions, these articles placed greater emphasis on how chronic hip pain affects movement performance, functional activities, physical activity, and participation‐related outcomes. Commonly reported outcomes included gait parameters [32], single‐leg squat performance [33, 34], pelvic kinematics [35], physical activity level [36], movement patterns [37, 38], and quality of life [39].

Most studies in this domain focused on activity‐level limitations. Skou et al. [34] and Ferrer‐Peña et al. [33] examined altered movement patterns in patients with chronic hip pain, including changes in gait, pelvic movement, hip adduction, hip internal rotation, and single‐leg functional tasks. These findings suggest that chronic hip pain is associated not only with pain intensity or structural abnormalities, but also with impaired movement control and reduced functional performance during daily activities.

Rehabilitation‐related studies further evaluated whether activity‐level impairments could be improved through movement‐based interventions. Smith and his colleagues [40] introduced a 12‐week rehabilitation program, as well as Palaniappan et al. [37] suggested interventions such as movement‐pattern training, movement‐pattern modification, cycling combined with education, structured exercise programs, and home‐based rehabilitation to improve pain, physical function, movement quality, and activity participation. These studies indicate that activity‐focused interventions may help translate symptom improvement into better functional performance.

Participation‐level outcomes were less frequently reported. A limited number of studies examined broader outcomes such as quality of life or thorough physical activity participation [39, 41]. Work participation, social role performance, and long‐term community engagement were rarely assessed. This suggests that although activity limitations have received increasing attention in chronic hip pain research, participation restrictions remain relatively underrepresented.

### 3.5 Personal Factors

A total of seven articles were classified under this domain, accounting for 17.9% of the studies included. These studies mainly examined individual characteristics and psychosocial factors associated with chronic hip pain, including psychological status [42, 43], sleep quality [44], race differences [45], and pain management strategies [46].

Psychological factors were the most frequently reported personal factors. Studies examined mostly depression and/or anxiety, or preoperative psychological status in relation to pain severity, postoperative pain persistence, and patient‐reported outcomes [42, 43, 47]. These findings suggest that psychological status may contribute to differences in pain experience and recovery among patients with chronic hip pain.

Other personal factors included sleep‐related characteristics, races, and individual pain management strategies. Sleep disturbance was reported by Tang et al. [44] in relation to pain severity and postoperative pain outcomes, while pain management behaviors were examined as factors associated with patients' self‐reported outcomes and long‐term recovery [46, 48]. These studies indicate that chronic hip pain outcomes may be influenced not only by structural pathology or physical impairment, but also by patient‐level characteristics.

### 3.6 Environmental Factors

Only two articles were classified under the ICF domain of environmental factors, accounting for 5.1% of the included studies. This was the least represented domain among the included literature. These studies mainly focused on care delivery models and external rehabilitation resources, including community‐based care and online education for people with hip or knee osteoarthritis‐related pain [49, 50].

Hurley et al. examined community‐based care for people with chronic knee and hip pain, reporting outcomes related to clinical improvement and healthcare utilization. Nelligan and his collegues investigated an online education approach, reflecting the potential role of digital resources in supporting knowledge, pain self‐efficacy, and self‐management. These studies suggest that environmental factors may influence how patients access rehabilitation, receive support, and manage chronic hip pain outside traditional clinical settings.

Overall, personal and environmental factors were substantially less studied than body‐level and activity‐level outcomes, suggesting that psychosocial and contextual dimensions of chronic hip pain remain underrepresented in the current literature.

### 3.7 Methodological Quality and Risk‐Of‐Bias Assessment

Among all the included studies, 38 completed studies were eligible for methodological quality or risk‐of‐bias assessment, while one study was a protocol and was not formally assessed because it did not report completed study results. The assessed studies included 6 individually randomized controlled trials, 1 cluster‐randomized controlled trial, 21 cohort studies, and 10 analytical cross‐sectional studies.

For the 6 individually RCTs, 3 studies were judged as having some concerns and 3 were judged as having a high risk of bias. The cluster‐randomized controlled trial, assessed using the RoB 2 tool for cluster‐randomized trials, was judged as having a high risk of bias.

Among the 21 cohort studies assessed using the JBI Critical Appraisal Checklist for Cohort Studies, 6 were considered high quality, 10 were considered moderate quality, and 5 were considered low quality.

Among the 10 analytical cross‐sectional studies assessed using the JBI Critical Appraisal Checklist for Analytical Cross‐Sectional Studies, 4 were considered high quality and 6 were considered moderate quality.

Overall, among the 38 completed studies formally assessed, 10 studies were considered high quality, 19 were considered moderate quality, and 9 were considered low quality (detailed information was listed in supplementary file 1). These findings indicate that although most included studies were suitable for evidence synthesis, the overall certainty of the conclusions should be interpreted with caution because several studies had a potential risk of bias, particularly related to confounding control, missing data, and outcome measurement.

## 4 Discussion

This scoping review synthesized 39 studies on chronic hip pain published between 2020 and 2025 using the ICF framework. Approximately half of the included studies focused on structures and functions, mainly addressing structural abnormalities, pain mechanisms, clinical assessment, and symptom‐oriented interventions. Fewer studies examined activity, participation, personal factors, or environmental influences. Overall, the findings indicate that current research remains largely centered on body‐level impairments and symptom management, while broader functional and contextual aspects of chronic hip pain are less consistently addressed.

### 4.1 ICF Framework Expands Assessment Beyond Structural Pathology

Traditional assessment of chronic hip pain often emphasizes imaging findings, pain intensity, range of motion, and muscle strength. These measures are essential for identifying structural abnormalities and body‐level impairments, but they may not fully explain patients' pain experience or functional disability. For example, Kim et al. reported that many hips with radiographic osteoarthritis were not painful, whereas many painful hips did not show radiographic osteoarthritis [6]. Similarly, in 2022, Ronda et al. found only a modest association between hip pain and radiographic hip osteoarthritis in early symptomatic hip OA [51]. As these findings were derived from observational analyses, they primarily provide evidence of an association and discordance between structural abnormalities and pain rather than definitive evidence.

These findings provide cautious support for the need for an assessment model that extends beyond structural pathology. Previous arthritis research has also suggested that the ICF provides a comprehensive framework for evaluating health impact at both individual and population levels [52]. However, direct evidence evaluating the clinical application of ICF‐based assessment specifically in chronic hip pain remains limited. In chronic hip pain, such an approach may help integrate structural findings with functional performance, psychosocial status, self‐management ability, and contextual barriers, thereby providing a more complete understanding of patients' rehabilitation needs.

### 4.2 ICF Framework Supports Comprehensive Diagnostic Reasoning

The ICF framework does not replace disease‐based diagnosis or anatomical classification. Instead, it complements them by linking structural findings to functional consequences and contextual influences. Based on the ICF framework [7] and previous evidence supported by ICF Core Set research in chronic pain and musculoskeletal conditions [53] we developed an ICF‐informed clinical reasoning table to illustrate how different domains may contribute to assessment, diagnostic interpretation, and treatment planning (Table 2).

**TABLE 2:** ICF‐based clinical reasoning framework for chronic hip pain.

ICF domain	Clinical problem	Assessment methods	Diagnostic implications	Treatment/management strategies
Structures and Functions	Pain, ROM limitation, muscle weakness, structural abnormalities, altered pain processing	Imaging, physical examination, PROMs, pain scales, ROM and strength testing, sensory testing	Identifies structural or physiological contributors to pain and distinguishes local pathology from pain‐related dysfunction	Medication, injection, nerve block, RFA/PRF, physical modalities, strengthening, pain modulation
Activity	Gait abnormality, movement dysfunction, walking limitation, difficulty with squatting or stair climbing	Gait analysis, functional performance tests, movement analysis, activity‐related pain assessment	Determines how body‐level impairments translate into functional limitations	Movement‐pattern training, gait retraining, functional exercise, home‐based rehabilitation [54]
Participation	Reduced work ability [55], limited physical activity participation, social role restriction, reduced quality of life	QoL scales, employment status, physical activity participation, social role assessment	Identifies the broader impact of chronic hip pain on social roles and meaningful life activities	Community‐based rehabilitation, return‐to‐work planning, long‐term physical activity promotion, participation‐focused management
Personal factors	Depression, anxiety, poor sleep, obesity, pain catastrophizing, low self‐efficacy	Psychological questionnaires, sleep assessment, BMI, pain coping measures, self‐efficacy scales	Explains individual differences in pain perception, prognosis, and treatment response	Education, CBT‐informed care [56], sleep management, weight management [57], lifestyle modification, self‐management support
Environmental factors	Limited healthcare access, inadequate social support, workplace demands [58], limited rehabilitation or community resources	Assessment of care access, social support, workplace context, assistive resources, digital health access	Identifies external barriers or facilitators influencing recovery and rehabilitation adherence	Environmental modification, digital rehabilitation, community resources, workplace adaptation, multidisciplinary care pathways

*Note:* This table was developed as an ICF‐informed clinical reasoning framework based on the findings of the present review. Strategies not directly examined in the included studies were derived from the ICF framework and supported by relevant external literature.

Abbreviations: BMI: body mass index; CBT: cognitive behavioral therapy; PRF: pulsed radiofrequency; PROM: passive range of motion; QoL: quality of life; RFA: radiofrequency ablation; ROM: range of motion.

An ICF‐based diagnostic approach may help clinicians move beyond the identification of a potential pain generator by linking chronic hip pain to its effects on functioning, daily activities, participation, and recovery‐related factors. The available evidence suggests that this multidimensional perspective may support more comprehensive and clinically meaningful diagnostic reasoning.

### 4.3 ICF Framework Guides Individualized Treatment and Rehabilitation

The findings of this review suggest that most treatment studies for chronic hip pain remain focused on body‐level impairments. Pharmacological treatments, nerve blocks, radiofrequency procedures, shockwave therapy, and other interventional techniques primarily aim to reduce pain or improve local physical dysfunction. However, the evidence supporting these interventions was methodologically variable: several randomized trials had some concerns or a high risk of bias, while many observational studies had limited control of confounding. Their reported benefits should therefore be interpreted as suggestive rather than definitive evidence of comparative effectiveness. These approaches may contribute to symptom control, but symptom reduction alone may not be sufficient to restore activity or participation.

Broader chronic musculoskeletal pain literature shows that biological, psychological, and social factors contribute to pain persistence [59], and integrated ICF‐behavioral models in osteoarthritis suggest that combining biomedical and behavioral perspectives may better explain disability. For chronic hip pain specifically, qualitative evidence suggests that patients' beliefs, expectations, and care experiences influence how they understand and manage their condition [4]. These findings support the use of ICF‐informed treatment planning, in which interventions are selected according to pathology, as well as to activity limitations, participation goals, psychological status, self‐management needs, and environmental barriers. Further high‐quality, hip‐specific studies are required to determine whether this multidimensional approach improves clinical outcomes.

### 4.4 Clinical Implications for Activity and Participation, and Other Factors

Although only a few of studies focused on activity and participation, personal factors, and environmental factors, these domains have important clinical implications for chronic hip pain management. From an activity and participation perspective, clinical assessment should not rely solely on imaging findings, pain intensity, range of motion, or muscle strength. It should also include walking ability, stair negotiation, sit‐to‐stand performance, squatting, physical activity level, work‐related demands, social participation, and quality of life [60]. These outcomes reflect the real‐world impact of chronic hip pain and may help clinicians identify treatment goals for patients. Recent recommendations for hip osteoarthritis also emphasize individualized, multicomponent non‐pharmacological management, including education, exercise, weight management when relevant, and support for long‐term adherence [61, 62].

Personal factors should also be considered during clinical reasoning and treatment planning. Psychological status, fear avoidance, self‐efficacy, sleep disturbance, lifestyle factors, and pain management behaviors may influence pain persistence, treatment adherence, and functional recovery [4, 42, 44, 59]. A recent umbrella review of chronic musculoskeletal pain reported that fear avoidance, poorer support networks, lower socioeconomic status, smoking, and high pain levels were associated with the development of chronic musculoskeletal pain [59]. In addition, qualitative evidence in people with chronic hip pain suggests that patients' beliefs and expectations about pain, aging, diagnosis, and care may shape how they interpret symptoms and engage with treatment [4]. Therefore, assessment of personal factors may help clinicians identify patients who need additional education or supporting strategies.

Environmental factors further indicate that chronic hip pain management should also consider community‐based exercise programs, digital education resources, family or social support, workplace demands, assistive devices, and healthcare delivery models, which may affect whether patients can engage in treatment and maintain long‐term functional improvement. For example, Hurley et al. found that community‐based rehabilitation programs for people with chronic knee and hip pain may support exercise participation, self‐management, and healthcare utilization outcomes [50]. Nelligan and his colleagues also suggested that digital education approaches have also been developed to improve osteoarthritis knowledge, pain self‐efficacy, exercise confidence, and self‐management behaviors in people with hip and/or knee osteoarthritis [49]. Because the environmental evidence was limited in quantity and included mixed hip and knee populations, these findings should be interpreted as indicating the potential relevance of environmental resources rather than establishing hip‐specific effectiveness. Nevertheless, they suggest that environmental resources and barriers should be considered when planning rehabilitation and long‐term management.

### 4.5 Strengths, Limitations and Future Directions

This review has several strengths. First, it used the ICF framework as a conceptual structure to classify and synthesize evidence on chronic hip pain. This design allowed the review to move beyond a purely pathology‐based or intervention‐based summary and to examine chronic hip pain across body functions and structures, activity and participation, personal factors, and environmental factors. Second, by classifying studies according to their primary ICF domain, the review was able to identify not only what evidence is available, but also where current evidence is concentrated or insufficient. This design therefore enabled the review to highlight the imbalance between body‐level research and the comparatively limited evidence on participation and environmental factors. Finally, the synthesis was explicitly linked to assessment, diagnostic reasoning, and treatment planning, which strengthens the clinical relevance of the review and supports translation of the ICF framework into chronic hip pain management.

However, several limitations should also be considered. First, some included studies addressed more than one ICF domain; therefore, classification according to the primary domain involved a degree of interpretation. Second, heterogeneity in study design, population, diagnosis, intervention, and outcome measures limited direct comparison across studies. Third, participation‐level and environmental evidence was limited, reducing the strength of conclusions regarding these areas. Last, some included studies involved mixed hip and knee populations and did not report separate hip‐specific findings.

Future studies should adopt more comprehensive outcome sets that capture not only pain, imaging findings, and physical impairment, but also real‐world function, psychosocial factors, and contextual barriers. Greater attention should be paid to work participation, return to social roles, long‐term community engagement, healthcare access, social support, digital rehabilitation, and community resources. Longitudinal studies are also needed to clarify how these factors interact over time and influence treatment response, self‐management, and long‐term recovery in people with chronic hip pain.

## 5 Conclusion

Current research on chronic hip pain remains predominantly focused on body‐level impairments and symptom management, whereas participation‐level outcomes and environmental factors are comparatively underrepresented. The ICF framework offers a useful structure for integrating structural findings with functional limitations, psychosocial factors, and contextual influences. Its application may support more comprehensive assessment, clinically meaningful diagnostic reasoning, and individualized treatment planning for people with chronic hip pain. However, conclusions regarding specific interventions and contextual factors should be interpreted cautiously due to the supporting evidence varying in methodological quality.

## Author Contributions

**Mingke You:** writing – original draft, conceptualization, methodology, formal analysis, resources, visualization. **Xue Zhang:** data curation, resources, investigation. **Kai Zhou:** conceptualization, methodology, supervision. **Jian Li:** supervision, funding acquisition, writing – review and editing. **Xi Chen:** supervision, formal analysis. **Gang Chen:** writing – review and editing, project administration, supervision, funding acquisition.

## Funding

The authors have nothing to report.

## Ethics Statement

The authors have nothing to report.

## Conflicts of Interest

The authors declare no conflicts of interest.

## Supporting information


**Supplementary file 1.** quality_assessment.

**Data S1:** PRISMA‐ScR_Fillable_Checklist.

## Data Availability

The data that supports the findings of this study are available in the Supporting Information of this article.
